# Postpartum Medicaid Use in Birthing Parents and Access to Financed Care

**DOI:** 10.1001/jamahealthforum.2025.1630

**Published:** 2025-06-27

**Authors:** Jonas J. Swartz, Ashley Lawson Avis, M. Kate Bundorf, Marisa Elena Domino

**Affiliations:** 1 Department of Obstetrics & Gynecology, Duke University School of Medicine, Durham, North Carolina; 2 Duke-Margolis Institute for Health Policy, Durham, North Carolina; 3 Cecil G. Sheps Center for Health Services Research, UNC Chapel Hill, Chapel Hill, North Carolina; 4 Sanford School of Public Policy, Duke University, Durham, North Carolina; 5 Center for Health Information and Research, College of Health Solutions, Arizona State University, Phoenix

## Abstract

**Importance:**

The American Rescue Plan of 2021 allowed states to expand pregnancy Medicaid coverage to 12 months post partum. How the new policy affects Medicaid coverage and health care utilization is largely unknown.

**Objectives:**

To quantify insurance coverage and care utilization for postpartum individuals under Medicaid policies that extended postpartum coverage to 12 months after delivery from 60 days.

**Design, Setting, and Participants:**

A retrospective study of Medicaid coverage and utilization in North Carolina using Medicaid claims from March 2016 to December 2023 was conducted. All Medicaid-funded births in North Carolina from January 2017 through December 2022 were included.

**Exposure:**

A total of 3 periods were differentiated: before the COVID-19 public health emergency (PHE), during the PHE when there was a moratorium on Medicaid disenrollment, and after North Carolina adopted the 12-month postpartum extension through the American Rescue Plan of 2021.

**Main Outcomes and Measures:**

Length and type of postpartum Medicaid enrollment were evaluated. Utilization outcomes included indicators of (1) the receipt of at least 1 postpartum visit; (2) any contraceptive visit; (3) any primary care visit; (4) any outpatient mental health care, and (5) any outpatient substance use disorder (SUD) care.

**Results:**

There were 353 957 Medicaid-funded births in North Carolina from January 2017 through December 2022. During the postpartum extension, Medicaid recipients were more likely to have been continuously covered by comprehensive Medicaid at 12 months post partum (97.1% vs 26.5% pre-PHE). Beneficiaries in the extended coverage cohorts were substantially more likely to use Medicaid-financed care than those in the pre-PHE cohort for contraception (47.8% for the PHE cohort and 47.9% for the extension cohort vs 38.0% for the pre-PHE cohort), primary care (68.1% for the PHE cohort and 71.4% for the extension cohort vs 25.3% for the pre-PHE cohort), mental health (22.1% for the PHE cohort and 25.7% for the extension cohort vs 7.5% for the pre-PHE cohort) and substance use disorder visits (3.6% for the PHE cohort and 5.3% for the extension cohort vs 2.2%for the pre-PHE cohort) within 12 months, although there was evidence of delays in early postpartum and contraceptive visits.

**Conclusions and Relevance:**

Results of this study suggest that extending Medicaid coverage for 12 months post partum was associated with expanded opportunities for greater access to Medicaid-financed medical and behavioral health care. Both prevention and ongoing treatment of chronic conditions may help mitigate key adverse outcomes. Findings may help policymakers and public health officials understand how extended coverage affects access to Medicaid-financed care.

## Key Points

**Question:**

Is 12 months of postpartum Medicaid coverage in North Carolina provided to birthing parents associated with greater access to Medicaid-financed health care?

**Findings:**

In this cohort study of 353 957 Medicaid-funded births in North Carolina, extended postpartum Medicaid coverage programs was associated with prolonged enrollment to at least 12 months for the more than 95% of affected beneficiaries. This facilitated covered visits for contraceptive care, primary care, mental health care, and substance use disorder.

**Meaning:**

In this study, a 12-month postpartum Medicaid coverage program was associated with improved Medicaid coverage and increased utilization of Medicaid-financed services that potentially could mitigate key adverse outcomes.

## Teaser

This cohort study assesses whether 12 months of postpartum Medicaid coverage in North Carolina provided to birthing parents was associated with greater access to Medicaid-financed health care.

## Introduction

Medicaid is the largest payer for births in the US covering more than 40% of births.^1^ Most states, however, have historically limited pregnancy-specific Medicaid coverage to approximately 60 days after delivery, leaving many beneficiaries without coverage in a time when they still had significant pregnancy-related mental and physical health needs. To address this coverage gap, the American Rescue Plan of 2021 allowed states to extend postpartum coverage for up to 12 months post partum beginning on April 1, 2022. To date, all states except Arkansas have implemented or plan to implement a full or partial extension.^2^ While it may be too early to assess the full effects of the 12-month postpartum extension, the federal COVID-19 Public Health Emergency (PHE) established a moratorium on disenrollment in Medicaid, effectively extending coverage for pregnant people well beyond the traditional 60-day period. This policy offers a window into the potential effects of extended Medicaid coverage for postpartum individuals.

Our study provides early evidence on the implications of postpartum coverage extensions by analyzing the experience of birthing parents in North Carolina. We differentiate among 3 time periods: before the public health emergency (pre-PHE) when post-partum coverage was limited to 60 days, during the public health emergency (PHE) when states were required to maintain coverage, and after North Carolina implemented the 12-month postpartum extension (extension). We document how Medicaid postpartum coverage differed across cohorts and then describe Medicaid-financed care utilization during 3 to 12 months post partum among the PHE cohort, including maternity as well as physical and behavioral health care.

We also examine differences among the 3 cohorts in their use of services in the 60-day postpartum period. We hypothesize that having coverage for a longer time period may affect the timing of care for services such as primary or even behavioral care, although the direction is not clear. For example, people with only 60 days of coverage may actively seek care during this period of low out-of-pocket-costs and extending coverage may decrease the urgency of using services during the 60-day postpartum period. For others, a short coverage window may discourage their use of services during the 60-day period if they felt they could not receive necessary follow-up care (such as for most behavioral health services). For this group, extended coverage may increase the use of services during the 60-day postpartum period. Finally, we compare 60-day service use for the pre-PHE cohort to 12-month service use in the extension cohorts to calculate the extent to which extended coverage is associated with overall Medicaid-financed service use.

## Methods

### Data and Study Sample

We used North Carolina Medicaid membership, claims, and encounter data from March 2016 through December 2023. Our study sample included Medicaid enrollees aged 14 through 48 years who had a Medicaid-funded birth between January 1, 2017, and December 31, 2022. The University of North Carolina institutional review board approved this study. The requirement for informed consent was waived under 45 CFR 46.116(d). We followed the Strengthening the Reporting of Observational Studies in Epidemiology (STROBE) reporting guideline.

### Insurance Coverage

We identified the Medicaid program that covered the birthing parent’s delivery hospitalization as the baseline eligibility category. The 12-month postpartum extension primarily affected those with pregnancy-specific Medicaid eligibility (called Medicaid for Pregnant Women [MPW] in North Carolina) and thus we focus most of our analyses on birthing parents eligible for MPW. Other relevant baseline categories included full Medicaid, which does not have time limits on postpartum coverage and emergency Medicaid. In North Carolina, MPW and full Medicaid covered the same set of benefits although they are distinct eligibility categories. Emergency Medicaid covered only obstetric delivery or emergency care and not antepartum or postpartum care. Family planning coverage was an eligibility category examined after delivery; it provides very limited coverage, only covering visits that increase access to reproductive services.^3^

### Policy Timing and Cohort Definition

On March 18, 2020, the beginning of the PHE, Congress established a moratorium on disenrollment from Medicaid, effectively extending coverage for pregnant people beyond 60 days. While the PHE ended in May 2023, North Carolina adopted and implemented a 12-month postpartum extension for MPW in April 2022, which conferred guaranteed 12-month coverage for those who gave birth on February 1, 2022, or later.^4^ While this change occurred during the PHE moratorium, it increased the certainty around postpartum coverage so we consider it a separate policy period. People who gave birth on or after April 1, 2022, were eligible for a guaranteed 12 months of coverage post delivery; in contrast, the length of the postpartum coverage period was uncertain for people who gave birth between March 18, 2020, and March 30, 2022, due to the uncertainty regarding when the PHE would end.

We identify 3 cohorts of beneficiaries differentiated by their eligibility for postpartum Medicaid coverage at the time of their delivery: (1) pre-PHE, who delivered between January 1, 2017, and December 31, 2019; (2) PHE, who delivered between January 1, 2020, and March 31, 2022, prior to the postpartum extension; and (3) extension, who delivered between April 1, 2022, and December 31, 2022. The second and third groups effectively had at least 12 months of coverage, but only the third group had certainty on the length of coverage. Although the PHE did not start until March 2020, coverage was retained for people who gave birth starting in January 2020, since their 60 days of coverage ended after the start of the PHE.

### Birth Identification

We identified births in Medicaid claims databased on *International Statistical Classification of Diseases and Related Health Problems, Tenth Revision (ICD-10)* diagnosis codes, *Current Procedural Terminology* codes, and Healthcare Common Procedure Coding System codes indicating labor and delivery (eTable 1 in Supplement 1). We coded the delivery date as the date on the professional claim (with a delivery code) during an inpatient stay. To identify distinct deliveries, we included deliveries that were more than 300 days apart. Pregnancy episodes included 280 days before to 12 months after obstetric delivery.

### Utilization Measures

Utilization outcomes included indicators of (1) the receipt of at least 1 postpartum visit; (2) any contraceptive visit; (3) any primary care visit; (4) any outpatient mental health care; and (5) any outpatient substance use disorder (SUD) care (eTables 1 and 2 in Supplement 1).

### Statistical Analysis

We first examined insurance coverage for those covered by Medicaid at time of delivery and at 2, 4, and 12 months post partum, both overall and by type of coverage at the time of delivery. We then characterized utilization during the first 2 months, 3 to 6 months and 7 to 12 months post partum among those who had MPW for delivery in the PHE cohort, stratifying the behavioral health outcomes by receipt of corresponding behavioral health services prior to delivery.

We also compared utilization in the first 60 days post partum among the PHE and extension cohorts compared with the pre-PHE cohort. To control for differences in the composition of the cohorts by utilization and to account for nonindependence in the presence of multiple births, we estimate generalized estimating equation models with a logit link of receipt of the outcomes during the first 2 months of coverage controlling for covariates. The key model covariates were the indicators for beneficiaries in the 2 extended cohorts, with the pre-PHE cohort as the referent category. We note that these indicators will pick up differences in the outcomes for each cohort due both to differences in policies among the cohorts (eg, extended coverage, COVID-19 mitigation policies such as mask wearing or telehealth) as well as changes in the composition of the cohorts not otherwise controlled for by included covariates (eg, potentially greater job loss during the PHE might have newly qualified some birthing parents for MPW coverage). We also included delivery month fixed effects to control for seasonal trends; an indicator of whether any part of the postpartum period occurred during the COVID-19 shelter-in-place orders (March-May 2020); an indicator for beneficiaries who transitioned to managed care during their postpartum period; and birthing parent characteristics including age at delivery, rural, race and ethnicity, insurance less than 1 month in the prenatal period, and prenatal diagnoses of mental health or SUD. Race and ethnicity were reported in Medicaid databased on enrollment files. Race and ethnicity are included as demographic characteristics and were studied given data on racial and ethnic disparities in access to care and outcomes. We conducted a sensitivity analysis excluding births during the stay-at-home orders (March-May 2020).

Finally, we compared overall utilization rates between the pre-PHE cohort with 2 months of coverage to utilization by the PHE and Extension cohorts with 12 months of coverage to estimate the additional Medicaid-financed service utilization that occurred during both types of extended coverage. Statistical significance was set at 2-sided *P* < .05. Data were analyzed using Stata version 17 (StataCorp) and SAS version 9.4 (SAS Institute).

## Results

There were 353 957 Medicaid-funded births in North Carolina from January 2017 through December 2022 (eTable 2 in Supplement 1). North Carolina’s Medicaid-covered birthing population is racially and ethnically diverse with 2.0% identified as Asian or Native Hawaiian or Other Pacific Islander, 1.4% American Indian, 32.8% Black, 58.2% White, 5.5% indicating multirace, and 0.1% unknown race. Twenty-two percent report Hispanic ethnicity. A total of 56.8% of births were to parents residing in an urban county, and 6.4% of birthing parents had less than 1 month of Medicaid prenatal coverage; 19.5% had received a prenatal mental health diagnosis and 10.0% had received a prenatal substance use disorder (SUD) diagnosis, with both rates increasing between pre-PHE and PHE and extension cohorts.

### Coverage Patterns

Prior to the PHE, 55.4% of deliveries were covered by full Medicaid, 32.7% by MPW, and 12.1% by emergency Medicaid (eFigure 1 in Supplement 1). Coverage rates were relatively stable across cohorts.

Among those with MPW at the time of delivery, pre-PHE beneficiaries frequently lost comprehensive Medicaid coverage by 4 months post partum (Figure; eFigures 2-4 in Supplement 1). While most remained covered by MPW at 2 months post partum, by 4 months post partum, 26.8% had no Medicaid coverage, 41.2% had only family planning Medicaid, and 22.9% had full Medicaid (eFigure 3 in Supplement 1). Beneficiaries with baseline in MPW coverage were more likely to retain comprehensive coverage in the PHE and Extension cohorts, with more than 95% of beneficiaries remaining in either full Medicaid or MPW through 12 months post partum.

**Figure.**
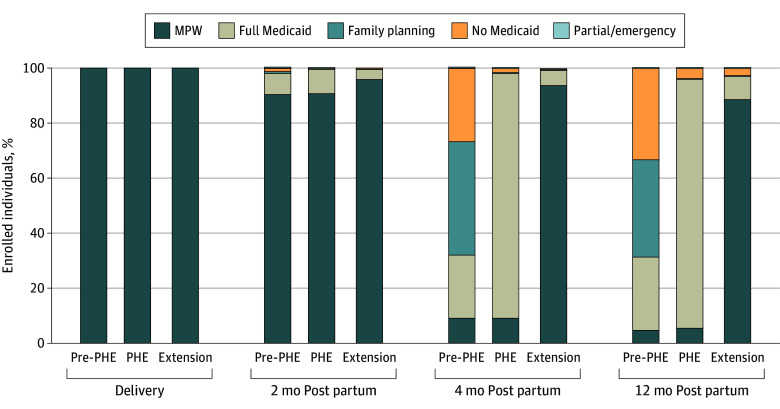
Coverage in the First Year Post Partum Among North Carolina Medicaid for Pregnant Women (MPW) Recipients by Policy Period (2017 Through 2022) Among deliveries to people with North Carolina MPW, coverage type at 3 postpartum time points is depicted. Policy periods are stratified as pre–COVID-19 public health emergency (pre-PHE), during the COVID-19 public health emergency (PHE), and after implementation of the 12-month postpartum extension (extension).

### Care Uptake With Expanded Postpartum Coverage

Many beneficiaries used Medicaid-funded care after 60 days of expanded postpartum coverage (Table 1). Among beneficiaries with MPW during the PHE period, 63.6% had a postpartum visit in the first 60 days after delivery. An additional 4.2% received their first postpartum visit in 3 to 6 months after delivery, and 1.4% received their first postpartum visit in 7 to 12 months after delivery.

**Table 1.** Utilization up to 1 Year Post Partum Among Beneficiaries With Medicaid for Pregnant Women^a^

Care	Medicaid-funded births, %						
	Pre-PHE (January 2017-December 2019) (n = 182 975 deliveries)	PHE (Jan 2020-March 2022) (n = 128 747 deliveries)			Extension (April 2022-December 2022) (n = 42 235 deliveries)		
	0-60 d	0-60 d	3-6 mo	7-12 mo	0-60 d	3-6 mo	7-12 mo
Covered at last month of each time period	100	100	100	100	100	100	100
Post partum							
Visit	69.8	63.6	16.4	9.1	59.0	17.6	10.6
Visit with prior visit^b^	NA	NA	12.2	7.7	NA	12.3	8.8
Visit without prior visit^b^	NA	NA	4.2	1.4	NA	5.3	1.8
Contraception							
Visits	38.0	32.3	26.9	19.7	31.2	27.9	21.4
Visits with prior visit^b^	NA	NA	15.4	15.4	NA	15.5	16.9
Visits without prior visit^b^	NA	NA	11.5	4.3	NA	12.4	4.5
PCP							
Visits	25.3	24.9	41.3	51.0	29.4	45.4	53.7
Visits without prior visit^b^	NA	NA	26.8	17.2	NA	26.6	15.9
MH							
Visits among beneficiaries with prenatal MH	26.9	27.8	31.4	38.0	31.8	34.1	40.1
Visits among beneficiaries with prenatal MH without prior visit^b^	NA	NA	15.3	12.5	NA	14.9	11.3
SUD							
Visits among beneficiaries with prenatal SUD	29.7	26.3	20.5	22..0	33.4	25.1	26.1
Visits among beneficiaries with prenatal SUD without prior visit^b^	NA	NA	3.9	3.9	NA	3.6	4.4

Abbreviations: MH, mental health; NA, not applicable; PCP, primary care practitioner; PHE, public health emergency; pre-PHE, pre-COVID 19 PHE; SUD, substance use disorder.

^a^ January 2020 through March 2022 encompassing 3 timeframes: pre-PHE vs PHE vs 12-month postpartum extension.

^b^ For 3-6 months, prior visit means a visit during the 0- to 60-day window. For 7-12 months, prior visit means a visit during the 0- to 60-day window or 3- to 6-month window. In the pre-PHE cohort, individuals with Medicaid for Pregnant Women had 60 days of postpartum coverage.

Contraceptive visits were most common in the first 2 postpartum months (32.3%), but 26.9% of birthing parents received contraception in 3 to 6 months post partum and 19.7% in 7 to 12 months post partum. Many of the later contraception visits were repeat visits, but 11.5% of beneficiaries had their first contraceptive visit at 3 to 6 months post delivery and 4.3% at 7 to 12 months post delivery.

Beneficiaries were increasingly likely to attend a PCP visit over time, with 25.3% receiving a PCP visit in 0 to 60 days, 41.3% in the 3 to 6 months, and 51.0% in 7 to 12 months after delivery. A total of 26.8% of birthing parents connected with a PCP for the first time at 3 to 6 months after delivery and 17.2% at 7 to 12 months after delivery.

Beneficiaries were found to have sustained mental health utilization in the extended coverage period. Among those with a prenatal mental health diagnosis, 27.8% had a mental health visit in the first 2 months, 15.2% had their first postpartum mental health visit 3 to 6 months; 12.4% had their first visit 7 to 12 months post partum. SUD visits followed a similar pattern, although utilization rates were highest during the first 60 days. Only 26.3% of beneficiaries with a prenatal SUD diagnoses had a postpartum visit for care that included a SUD diagnosis in the first 60 days; 3.9% received their first SUD visit during 3 to 6 months post partum, and an additional 3.9% received an SUD visit 7 to 12 months post partum.

### Cross-Cohort Comparison of Utilization During the 60-day Postpartum Period

The extent to which 60-day utilization differed across the MPW cohorts varied by type of service (Table 2). After adjusting for patient characteristics, postpartum visit attendance within 60 days was lower in the PHE cohort (percentage point difference, −5.84; 95% CI, −6.52 to −5.16) and lower in the extension cohort (percentage point difference, −7.39; 95% CI, −8.67 to −6.11) compared with the pre-PHE cohort. Similarly, rates of contraception visits within 60 days were lower in the PHE cohort (percentage point difference, −5.99; 95% CI, −6.68 to −5.30) and lower in the extension cohort (percentage point difference, −6.76; 95% CI, −8.03 to −5.49) compared with the pre-PHE cohort. PCP visit rates within 60 days were lower in the PHE cohort (percentage point difference, −1.07; 95% CI, −1.70 to −0.44) and the extension cohort (percentage point difference, 0.73; 95% CI−0.44 to 1.90) compared with pre-PHE cohort. Among those with a prenatal mental health diagnosis, the probability of having a mental health visit with 60 days post partum was greater for the extension cohort than the pre-PHE cohort (percentage point increase, 6.81; 95% CI, 3.95-9.67). Similarly, the frequency of postpartum SUD visits during the first 60 days was greater for the extension than the pre-PHE cohort both overall and among those with a prenatal SUD diagnosis. Differences between the PHE and pre-PHE cohorts in substance use visits were not statistically significant. Results were similar in sensitivity analyses that excluded births from March through May 2020, suggesting that our results are not explained by pandemic-era care disruptions (eTable 3 in Supplement 1).

**Table 2.** Adjusted Difference by Cohort in Care Utilization in the First 60 Days Post Partum Compared With Pre-PHE^a^

Care	AME^b^ (95% CI)	
	PHE	Extension
Postpartum visit	−5.84 (−6.52 to −5.16)^c^	−7.39 (−8.67 to −6.11)^c^
Contraception visit	−5.99 (−6.68 to −5.30)^c^	−6.76 (−8.03 to −5.49)^c^
PCP visit	−1.07 (−1.70 to −0.44)^c^	0.73 (−0.44 to 1.90)
Mental health visit	1.02 (0.65 to 1.39)^c^	2.62 (1.87 to 3.36)^c^
Mental health visit in subpopulation with a prenatal mental health diagnosis	1.63 (0.01 to 3.24)^c^	6.81 (3.95 to 9.67)^c^
SUD visit^d^	0.02 (−0.16 to 0.20)	1.64 (1.23 to 2.05)^c^
SUD visit in subpopulation with a prenatal SUD diagnosis^e^	−0.52 (−2.86 to 1.81)	12.56 (8.49 to 16.64)^c^

Abbreviations: AME, average marginal effects; PCP, primary care practitioner; PHE, public health emergency; SUD, substance use disorder.

^a^ Covariates: cohort indicators (with the pre-PHE cohort as the referent category), delivery month, COVID-19 shelter-in-place orders (March-May 2020), an indicator for beneficiaries who transitioned to managed care during their postpartum period, and birthing parent characteristics including age at delivery, rural, race and ethnicity, insurance less than 1 month in the prenatal period, and prenatal diagnoses of mental health or SUD.

^b^ AME is the percentage point difference in the likelihood of using any care of the given type relative to the pre-PHE cohort. Sample includes North Carolina Medicaid beneficiaries with Medicaid for Pregnant Women at delivery who have coverage at 60th day post partum.

^c^ Statistically significant at the *P* < .05 level.

^d^ Excludes Unknown race due to perfect prediction. That is, the few individuals with unknown race had the same SUD visit outcome, so the model is unable to determine the effect of this covariate.

^e^ Excludes Unknown race and Asian or Hawaiian or Other Pacific Islander due to perfect prediction. That is, there is no variation in the outcome for individuals in these racial categories so they were excluded from the list of covariates for this model.

While extended coverage was associated with lower use of some types of Medicaid-financed services within 60 days, extended coverage was associated with greater overall use of Medicaid-financed care for most types of services within 12 months post partum. For postpartum visits, 60-day rates for the pre-PHE cohort (69.8%) were virtually identical to 12-month rates for the PHE cohort (69.2%) and slightly higher than 12-month rates for the extension cohort (66.1%) (Table 3). For most of the remaining outcomes, beneficiaries in the extended coverage cohorts were substantially more likely to use Medicaid-financed care than those in the pre-PHE cohort for contraception (47.8% for the PHE cohort and −47.98% for the extension cohort vs 38.0% for the pre-PHE cohort), primary care (68.1% for the PHE cohort and −71.4% for the extension cohort vs 25.3% for the pre-PHE cohort), mental health (22.1% for the PHE cohort and −25.76% for the extension cohort vs 7.58% for the pre-PHE cohort), and substance use disorder visits (3.64% for the PHE cohort and −5.3% for the extension cohort vs 2.2%for the pre-PHE cohort) within 12 months (Table 3). Rates of SUD visits among those with a prenatal SUD diagnosis within 12 months among the extended coverage cohorts were also greater than those with 60 days of coverage.

**Table 3.** Unadjusted Rates of Service Use in 60 Days Post Partum^a^

Care	Medicaid-financed service use, %		
	Pre-PHE (2 mo)	PHE (12 mo)	Extension (12 mo)^b^
Postpartum visit	69.8	69.2	66.1
Contraception visit	38.0	47.8	47.9
PCP visit	25.3	68.1	71.4
Mental health visit	7.5	22.1	25.7
Mental health visit among beneficiaries with prenatal	26.9	55.0	57.6
SUD visit	2.2	3.6	5.3
SUD visit among beneficiaries with prenatal SUD	29.7	34.0	41.3

Abbreviations: PCP, primary care practitioner; PHE, public health emergency; pre-PHE, pre–COVID 19 PHE; SUD, substance use disorder.

^a^ Analysis is conditional on enrollment at 60 days for each cohort.

^b^ Extension cohort only includes individuals with 12 months of postpartum data.

However, across all 3 cohorts, less than half of those with a prenatal SUD diagnosis received postpartum SUD care (ranging from 29.7% for pre-PHE cohort to 41.3% for the extension cohort). Results remained consistent when we excluded births while stay-at-home orders were in effect (March 2020 to May 2020) or the first 2 months of the policy adjustment.

## Discussion

In this cohort study, the PHE restrictions on coverage redetermination and the postpartum coverage extension were associated with an increase in the continuity of Medicaid coverage for people giving birth. Pre-PHE, 7 of 10 lacked comprehensive Medicaid coverage by 4 months post partum; during the extension period, 97% of birthing parents kept comprehensive Medicaid through 12 months after delivery. Increased insurance continuity is important and has also been associated with reduction in overall and racial disparities in maternal mortality.^5,6^ These results may be useful for policymakers, administrators, and clinicians to meet service demands.

The results are consistent with studies of the effects of the Affordable Care Act on Medicaid coverage among birth people as well as evidence from the Pregnancy Risk Assessment Monitoring System (PRAMS) during the PHE.^5,6,7,8^ However, PRAMS data did not demonstrate evidence of improved outcomes such as contraceptive use, breastfeeding, or depression symptoms.^8^ Our study documents that Medicaid beneficiaries with extended coverage frequently accessed Medicaid-financed care in the 3 to 12 postpartum months, including postpartum follow up, contraception, PCP visits, and behavioral health services, with some beneficiaries using important postpartum services after 60 days.

We found some evidence of care delays when people had access to coverage for a longer time period. Expanded postpartum coverage was associated with decreased postpartum visits and contraceptive visits in the first 60 days post partum for the PHE and Extension cohorts relative to pre-PHE cohort (Table 2). However, overall utilization of postpartum visits over 12 months for both post-PHE cohorts was similar to 60-day utilization for the pre-PHE cohort (Table 3). We do note a modest correlation with lower rates of postpartum visits within 12 months among the extension cohort at a time when access to care should have been increasing due to the end of the pandemic and greater certainty about insurance coverage. One hypothesis is that we may be observing a trade-off between visit and clinician types during the extension if patients are seeing primary care practitioners who do not code the service as a postpartum screening visit (Table 3). Patients may also have had difficulty obtaining care even though they had coverage.

While initiation of contraception is recommended prior to resumption of intercourse to promote healthy birth spacing and reproductive autonomy,^9^ many birthing parents may not start contraception in this timeframe or may change contraceptive type. We find evidence of substantive Medicaid-financed contraceptive use during the extended postpartum coverage period. Sixteen percent of beneficiaries had their first contraceptive visit outside the 60-day timeframe and 31% had 1 or more follow up visits, which can be an opportunity to switch contraceptive types to a modality more appropriate with their longer term health status.^10,11^

We find extended coverage was associated with more mental health service use during the 60-day postpartum period, particularly among those who were previously diagnosed, and also more use of Medicaid-financed mental health services after 60 days. These findings warrant further examination as it could be associated with the policy change or with observed postpandemic population-level increases in mental health diagnoses.^12^ These results suggest that extended postpartum coverage could help address important postpartum mental health challenges. Suicide and behavioral health conditions accounted for one-third of North Carolina’s pregnancy-related deaths in 2018-2019, making them the most prevalent cause of death, as is true nationally.^13,14^ Treatment of substance use disorder deserves particular attention given state and national increasing trends in pregnancy-associated death from drug overdose.^14,15^

### Limitations

A key study limitation is the lack of information on services not financed by Medicaid, including possible undercounting of prenatal care visits or post-pregnancy care.^16,17^ Increases in Medicaid-financed services may not necessarily translate to increases in the use of any service, particularly if people would have transitioned to commercial insurance after 60 days. Evidence using PRAMS data, however, suggests that around 20% of individuals with Medicaid-financed births obtain commercial insurance post partum, which slightly declined following implementation of extended coverage, suggesting that health care use covered by alternative coverage sources may have been minimal.^8,17^ Additionally, it is possible in this descriptive study that observed changes in utilization may be due to factors unrelated to insurance coverage, such as changes in availability of care during the PHE. It is also important to consider the North Carolina context when interpreting the findings. North Carolina expanded Medicaid coverage in December 2023, after our study period. In addition, MPW coverage in North Carolina confers full Medicaid benefits, unlike other states that may restrict covered benefits to pregnancy-related care.^18^ The study period includes the July 2021 implementation of Medicaid Managed Care in NC. While this may have disrupted or consolidated care networks, our analysis controls for managed care implementation. Finally, both coverage and utilization are intermediate outcomes; future research should more directly assess beneficiary health.

## Conclusions

In this study, we observed substantial utilization of Medicaid-financed care among birthing parents in North Carolina following the implementation of extended Medicaid postpartum coverage. While we find some evidence this policy change was associated with delayed postpartum and contraceptive visits during extended coverage, the policy was overall associated with a substantial increase in Medicaid-financed care including postpartum care, contraception, primary care, behavioral health care and treatment of SUD.

## Notes

Supplement 1. **eTable 1.** Visit Definitions **eMethods.** Supplementary Methodology for Visit Identification **eTable 2.** Demographic Characteristics **eFigure 1.** Medicaid Coverage Category at Delivery **eFigure 2.** Coverage Over Time Among Those With MPW at Delivery, by Cohort Pre-PHE (1/2017-12/2019) **eFigure 3.** Coverage Over Time Among Those With MPW at Delivery, by Cohort PHE (1/2020-3/2022) **eFigure 4.** Coverage Over Time Among Those With MPW at Delivery, by Cohort Extension (4/2022-12/2022). **eTable 3.** Sensitivity Analysis of Adjusted Average Marginal Effects of Cohort on Care Utilization Outcomes in the First 60 Days Postpartum With Those Who Delivered in March 2020 – May 2020 Removed

Supplement 2.
Data Sharing Statement
