# Supplementary material for: Postpartum Medicaid Use in Birthing Parents and Access to Financed Care
Source: JAMA Health Forum. 2025 Jun 27;6(6):e251630. doi: 10.1001/jamahealthforum.2025.1630 (PMC12205403; doi:10.1001/jamahealthforum.2025.1630)
Supplement: Supplement 1. — eTable 1. Visit Definitions eMethods. Supplementary Methodology for Visit Identification eTable 2. Demographic Characteristics eFigure 1. Medicaid Coverage Category at Delivery eFigure 2. Coverage Over Time Among Those With MPW at Delivery, by Cohort Pre-PHE (1/2017-12/2019) eFigure 3. Coverage Over Time Among Those With MPW at Delivery, by Cohort PHE (1/2020-3/2022) eFigure 4. Coverage Over Time Among Those With MPW at Delivery, by Cohort Extension (4/2022-12/2022). eTable 3. Sensitivity Analysis of Adjusted Average Marginal Effects of Cohort on Care Utilization Outcomes in the First 60 Days Postpartum With Those Who Delivered in March 2020 – May 2020 Removed [file jamahealthforum-e251630-s001.pdf]

Supplemental Online Content

Swartz JJ, Avis A, Bundorf MK, Domino ME. Postpartum Medicaid use in birthing parents and access to financed care. *JAMA Health Forum.* 2025;6(6):e251630. doi:10.1001/jamahealthforum.2025.1630

eTable 1. Visit Definitions

eMethods. Supplementary methodology for visit identification

eTable 2. Demographic Characteristics

eFigure 1. Medicaid Coverage Category at Delivery

eFigure 2. Coverage Over Time Among Those With MPW at Delivery, by Cohort Pre-PHE (1/2017-12/2019)

eFigure 3. Coverage Over Time Among Those With MPW at Delivery, by Cohort PHE (1/2020-3/2022)

eFigure 4. Coverage Over Time Among Those With MPW at delivery, by Cohort Extension (4/2022-12/2022).

eTable 3. Sensitivity Analysis of Adjusted Average Marginal Effects of Cohort on Care Utilization Outcomes in the First 60 Days Postpartum With Those Who Delivered in March 2020 – May 2020 Removed

This supplemental material has been provided by the authors to give readers additional information about their work.

© 2025 Swartz JJ et al. *JAMA Health Forum*.

eTable 1. Visit Definitions

Category	Diagnosis Codes	CPT/HCPCS Codes	Other Codes/Notes
Delivery	O80, O82, Z37, Z390	Vaginal CPT Codes: 59400, 59409, 59410, 59610, 59612, 59614<br>Cesarean CPT Codes: 59510, 59514, 59515, 59618, 59620, 59622	HEDIS Inpatient stay revenue codes to identify inpatient stays associated with the delivery.<br>When a professional claim fell within the window of an inpatient stay, the date on the professional claim was used as the delivery date.<br>If no professional claim fell within the window of an inpatient stay, but there was a delivery diagnosis or CPT code present, the admission date was used as the delivery date.<br>Deliveries were collapsed within 300 days.
Contraception Visit	Injectable, IUD, Implant, Ring, Oral Pill, Patch, Diaphragm, Sterilization, and LARC Diagnosis Codes	Injectable, IUD, Implant, Ring, Oral Pill, Patch, Diaphragm, Sterilization, and LARC CPT/HCPCS Codes	NDCs for contraception<br>HEDIS Codes:<br>1. CCP-C Most/Moderately Effective Contraceptive Method<br>2. CCP-D Long-Acting Reversible Contraception Method
Postpartum Visit	Z391, Z392	0503F, S0281, 59430	1. Visits included when there was no diagnosis code for a postpartum visit but where a physician placed contraception (IUD, implant, injection).<br>2. Telehealth visits may be included if billed with modifier codes though no specific telehealth procedure codes were included.

© 2025 Swartz JJ et al. *JAMA Health Forum*.

PCP Visit	NA	Requires one of these 3 options:<br>1. Subset of E&M Codes<br>2. CPT Codes for bundled antepartum care NOT delivered by an OBGYN (identified by taxonomy codes)<br>3. T1015	Requires one of these 2 options:<br>1. Taxonomy code to indicate primary care provider*<br>2. Provider was ever an AMH

* Primary care provider taxonomy groups include: Community/Behavioral Health, Family Medicine, General Practice, Internal Medicine, OBGYN, Pediatrics, Clinic/Center, Multi-Specialty, Single Specialty, Nurse Practitioner, Physician Assistant

eMethods. Supplementary methodology for visit identification

We identified postpartum visits through a specific bonus payment for postpartum screening, visits with postpartum diagnoses, and visits for outpatient, clinician-placed contraception such as an intrauterine device, injection, or contraceptive implants at least three days after delivery. See technical appendix for specific codes. We examined the number of beneficiaries who had a bundled or global payment code that included postpartum care (e.g CPT 59410, Vaginal delivery and postpartum care) but did not separately have evidence of a postpartum care visit from analysis as we might not observe the presence or absence of a specific outpatient postpartum visit for these individuals. This group was less than 5% of the full sample, so we retained them for analysis, although this may artificially lower the postpartum care rate.

Contraceptive care was identified with procedure (CPT and HCPCS) codes, diagnosis codes, surgical procedure codes, and national drug codes for contraceptive prescriptions.(9) Primary care visits were identified using Evaluation and Management procedure codes combined with provider taxonomy code for primary care providers or health centers. This algorithm counts OB/Gyns services as primary care for claims using general office visit codes or if the OB/Gyn was recognized as an Advanced Medical Home.(10) Mental health and SUD visits were identified as any visit with one or more diagnosis codes for mental health or SUD. SUD diagnoses excluded tobacco use disorder.

© 2025 Swartz JJ et al. *JAMA Health Forum*.

eTable 2. Demographic Characteristics

Characteristic	Overall	Pre-PHE (Jan 2017-Dec 2019)	PHE (Jan 2020. – March 2022)	Extension (April 2022 – Dec 2022)	P-value
Deliveries (N)	353,957	182,975	128,747	42,235	
Age 14-48, mean (SD)	26.8 (5.8)	26.6 (5.8)	27.0 (5.8)	27.2 (5.9)	<0.001
Age 14-48, median [Q1 - Q3]	26 [22 - 31]	26 [22 - 30]	26 [22 - 31]	27 [23 - 31]	
Race - White only	206,030 (58.2%)	105,895 (57.9%)	74,883 (58.2%)	25,252 (59.8%)	
Race - Black only	116,000 (32.8%)	60,132 (32.9%)	42,425 (33.0%)	13,443 (31.8%)	<0.001
Race - Asian or Haw Pacific only	7,194 (2.0%)	3,772 (2.1%)	2,604 (2.0%)	818 (1.9%)	
Race - American Indian only	4,950 (1.4%)	2,496 (1.4%)	1,875 (1.5%)	579 (1.4%)	
Race - Multi-Race	19,421 (5.5%)	10,561 (5.8%)	6,784 (5.3%)	2,076 (4.9%)	
Race - Unknown	362 (0.1%)	119 (0.1%)	176 (0.1%)	67 (0.2%)	
Hispanic	79,283 (22.4%)	38,540 (21.1%)	29,689 (23.1%)	11,054 (26.2%)	<0.001
Urban counties	201,208 (56.8%)	103,288 (56.4%)	73,449 (57.0%)	24,471 (57.9%)	<0.001
Less than 1m prenatal coverage	22,702 (6.4%)	10,926 (6.0%)	8,652 (6.7%)	3,124 (7.4%)	<0.001
Mental Health diagnosis during pregnancy	69,191 (19.5%)	33,440 (18.3%)	26,646 (20.7%)	9,105 (21.6%)	<0.001
Substance use disorder diagnosis during pregnancy	35,224 (10.0%)	17,356 (9.5%)	13,579 (10.5%)	4,289 (10.2%)	<0.001

Caption: Among deliveries to people with NC Medicaid for Pregnant Women, demographic characteristics are listed. Policy periods are stratified as pre-COVID 19 public health emergency (Pre-PHE), during the COVID-19 public health emergency (PHE), and after implementation of the 12 month postpartum extension (Extension).

© 2025 Swartz JJ et al. *JAMA Health Forum*.

eFigure 1: Medicaid Coverage Category at Delivery

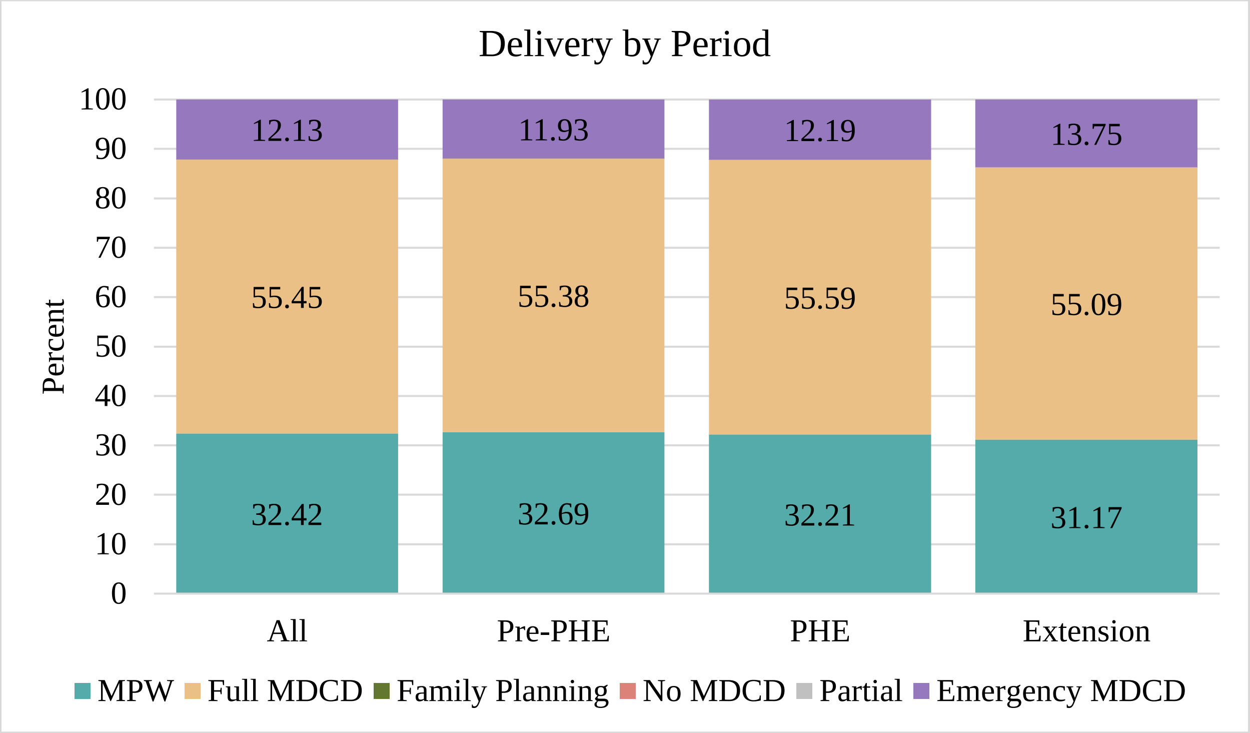

Caption: Among people with Medicaid coverage in NC from 2017 through 2022, figure depicts category of Medicaid listed at time of delivery. Policy periods are stratified as pre-COVID 19 public health emergency (Pre-PHE), during the COVID-19 public health emergency (PHE), and after implementation of the 12 month postpartum extension (Extension).

© 2025 Swartz JJ et al. *JAMA Health Forum*.

eFigure 2. Coverage Over Time among those with MPW at delivery, by Cohort

Appendix Figure 2

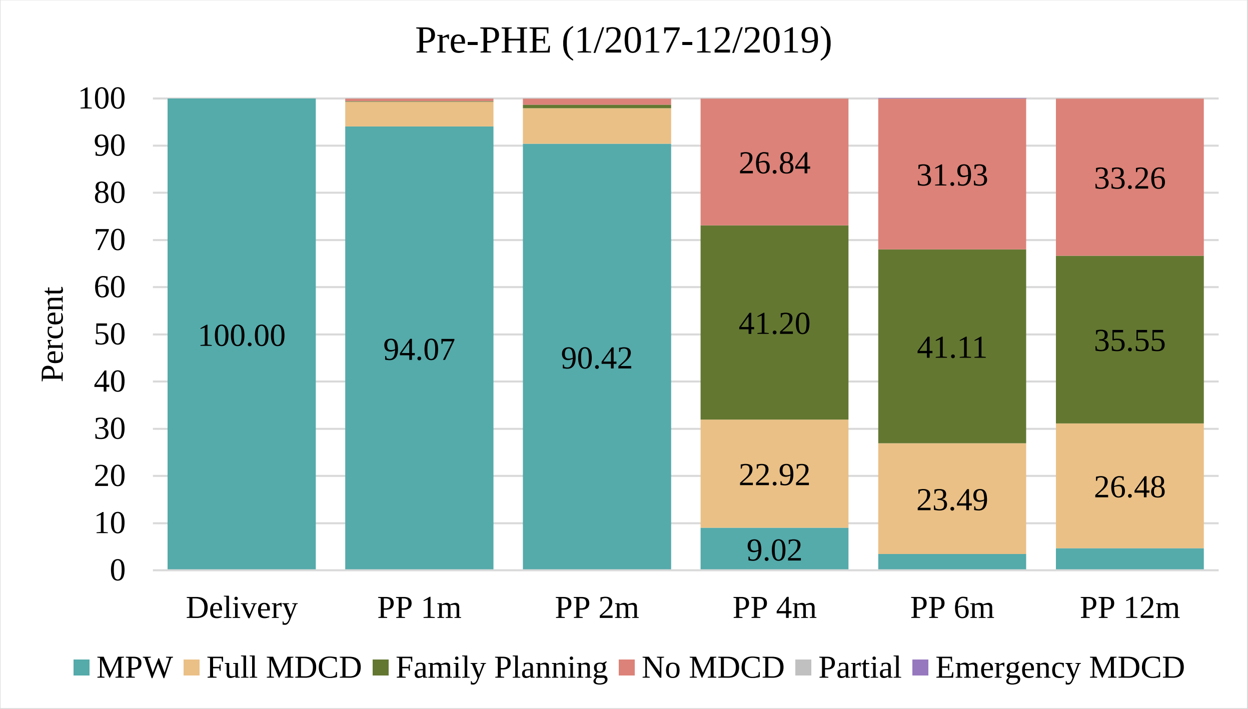

Appendix Figure 3. Coverage Over Time among those with MPW at delivery, by Cohort

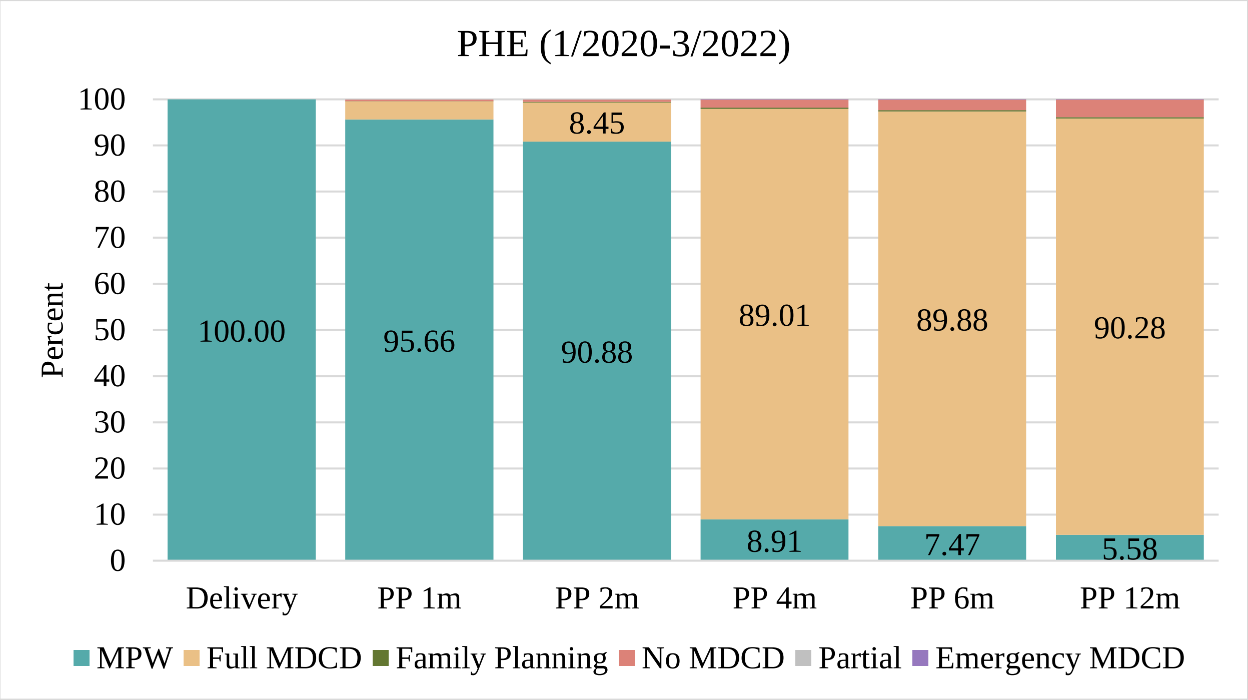

© 2025 Swartz JJ et al. *JAMA Health Forum*.

eFigure 4. Coverage Over Time among those with MPW at delivery, by Cohort

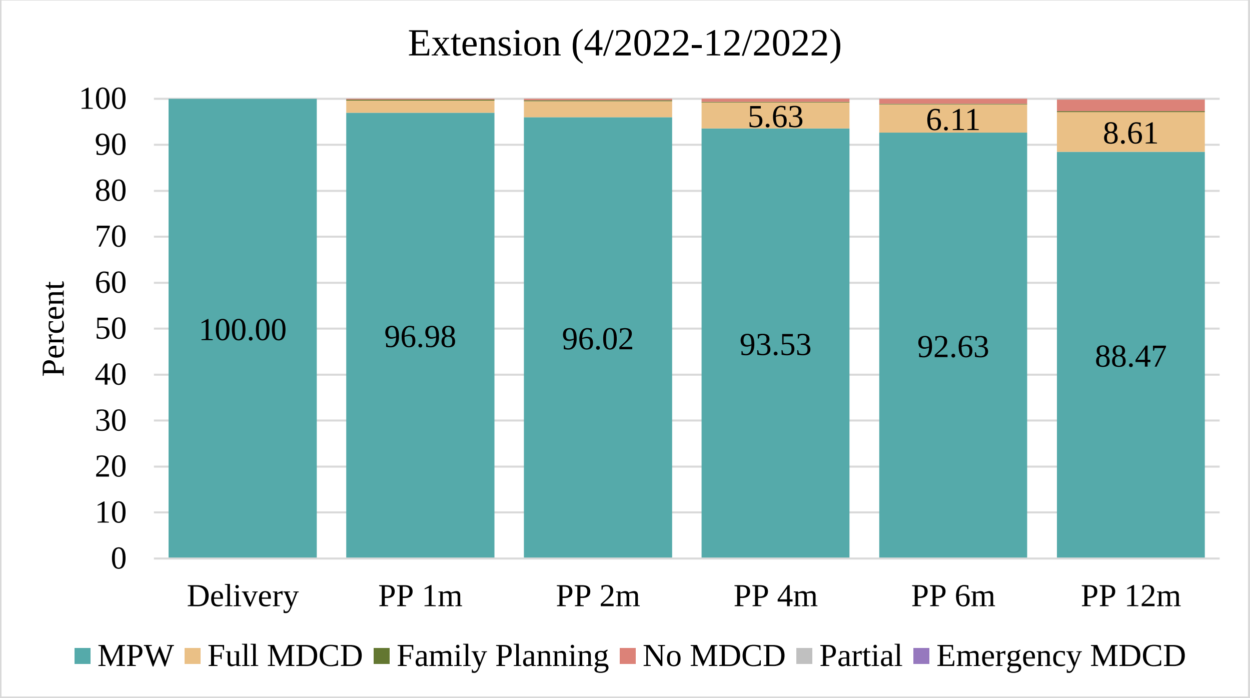

Caption: Detailed data at multiple timepoints of postpartum coverage among individuals delivering with NC Medicaid for Pregnant Women between 2017 and 2022. Policy periods are stratified as pre-COVID 19 public health emergency (Pre-PHE, Appendix Figure 2), during the COVID-19 public health emergency (PHE, Appendix Figure 3), and after implementation of the 12 month postpartum extension (Extension, Appendix Figure 4).
MPW- Medicaid for Pregnant Women
MDCD - Medicaid

© 2025 Swartz JJ et al. *JAMA Health Forum*.

eTable 3: Sensitivity Analysis of Adjusted Average Marginal Effects of cohort on care utilization outcomes in the first 60 days postpartum with those who delivered in March 2020 – May 2020 removed

	PHE		Extension	
**Care**	**AME**	**SE**	**AME**	**SE**
Postpartum visit	-5.84	0.35	-7.28	0.65
Contraception visit	-5.96	0.35	-6.79	0.65
PCP visit	-1.08	0.32	0.82[NS]	0.60
Mental Health visit*	1.02	0.19	2.61	0.38
Mental Health Visit in subpopulation with a Prenatal Mental Health diagnosis*	1.64	0.83	6.81	1.46
SUD visit*	0.02[NS]	1.19	1.64	0.21
SUD Visit in subpopulation with a Prenatal SUD diagnoses**	-0.52[NS]	1.25	12.54	2.08

* Excludes Unknown race due to perfect prediction
** Excludes Unknown race and Asian/Hawaiian Pacific due to perfect prediction
PCP- Primary Care Provider
SUD – Substance use disorder

© 2025 Swartz JJ et al. *JAMA Health Forum*.
