# Supplementary material for: Postpartum Medicaid Use in Birthing Parents and Access to Financed Care
Source: JAMA Health Forum. 2025 Jun 27;6(6):e251630. doi: 10.1001/jamahealthforum.2025.1630 (PMC12205403; doi:10.1001/jamahealthforum.2025.1630)
Supplement: Supplement 2. — Data Sharing Statement [file jamahealthforum-e251630-s002.pdf]

Data Sharing Statement

Swartz. Postpartum Medicaid Use in Birthing Parents and Access to Financed Care. *JAMA Health Forum*. Published June 27, 2025. doi:10.1001/jamahealthforum.2025.1630

Data
Data available: No

Additional Information
Explanation for why data not available: Data use agreements with the state of NC prevent us from sharing data.
